# Effects of Pomegranate Flower Extracts on Antioxidant Properties, Phenolic Content, and Quality Attributes of Nitrite Reduced Chicken Sausages

**DOI:** 10.1111/asj.70039

**Published:** 2025-02-13

**Authors:** Cem Okan Özer, Ganime Beyzanur Var, Ezgi Demir Özer, Birol Kiliç

**Affiliations:** ^1^ Faculty of Engineering and Architecture, Department of Food Engineering Nevsehir Hacı Bektas Veli University Nevsehir Turkey; ^2^ School of Applied Sciences, Department of Gastronomy and Culinary Arts Cappadocia University Nevsehir Turkey; ^3^ Faculty of Engineering, Department of Food Engineering Süleyman Demirel University Isparta Turkey

**Keywords:** bioactive content, chicken sausages, lipid oxidation, nitrite replacement, pomegranate flower extracts

## Abstract

The objective of present study was to examine the potential use of pomegranate flower extracts (PFEs) obtained from water and ethanol at antimicrobial doses in nitrite‐reduced chicken sausages. The impact of the extracts on the physicochemical, textural, and sensory attributes of the sausages was evaluated using both reduced doses of nitrite and a nitrite‐free preparation. The results revealed that incorporating PFE resulted in a sixfold and twofold enhancement in total phenolic content and antioxidant activity, respectively. The DPPH radical scavenging activity in sausages produced with ethanol‐based PFE was more than twice as high as in sausages treated with sodium nitrite. Additionally, the combination of low‐dose nitrite with PFE showed a reduction in TBARS values, leading to the prevention of lipid oxidation. Sausages with PFE exhibited lower pH and redness values, while maintaining similar aroma and odor characteristics as the controls. Although there was a slight negative impact on texture, color, and overall acceptability, PFE can be considered a potential alternative for improving bioactive content and oxidative stability of low‐nitrite chicken sausages. The application of PFE could offer a promising opportunity to enhance the nutritional profile and quality attributes of processed meat products.

## 1 Introduction

In recent years, there has been a significant trend towards minimally processed and natural products due to increased awareness of health and nutrition, concerns about carcinogens, and ethical and environmental considerations (McClements 2023). In particular, the use of synthetic additives such as nitrites, artificial antioxidants, and colorants in processed meat products has raised concerns about potential health‐related issues. These issues include the formation of carcinogenic nitrosamines and other toxicological effects. The additives are commonly used to preserve microbial quality, improve color and flavor, and prevent oxidative reactions in meat products. However, their safety has become an important topic of discussion (Serdaroğlu et al. 2023; Zhang et al. 2023). Excessive consumption of these additives has been associated with an increased risk of carcinogenesis, as well as the onset of allergic reactions, migraines, hyperactivity, dermal reactions, and digestive disturbances (Estévez 2021; Zhang et al. 2023).

Synthetic additives, including antioxidants and colorants, have raised significant health concerns and have restricted the consumption of meat products (Karre, Lopez, and Getty 2013; Shahidi and Ambigaipalan 2015). This growing concern has led to an increase in consumer demand and scientific research aimed at finding natural alternatives, particularly from plant sources, that can provide similar antioxidant and coloring properties (Banerjee et al. 2012; Devatkal, Kumboj, and Paul 2014; Manessis et al. 2020; Oswell, Thippareddi, and Pegg 2018; Shah, Bosco, and Mir 2014). The use of plant powders and extracts, including those derived from spices, herbs, fruits, and their by‐products, has been demonstrated to inhibit oxidation, enhance color, and improve aroma, thereby promoting their application on an industrial scale (Awad et al. 2022). Among these, fruits such as pomegranate, citrus, grapes, and vegetables with high antioxidant content have been the subject of considerable research and attention (Awad et al. 2022). In particular, utilization of pomegranate and its by‐products in food has increased in recent years due to the discovery of their high bioactive properties (Cheng et al. 2023; Das et al. 2021; García et al. 2021; Mo et al. 2022).

Pomegranate ( *Punica granatum* L.) is grown in various parts of the world (Conidi, Drioli, and Cassano 2020; Ge et al. 2021; Suman and Bhatnagar 2019). The flowers, leaves, stems, fruits, bark, and roots of the pomegranate plant contain a variety of potential bioactive compounds such as phenolic compounds, tannins, flavonoids, and complex polysaccharides (Das et al. 2021). Indeed, there has been an increasing interest in pomegranate by‐products recently due to their confirmed health benefits, including antimicrobial, antioxidant, antiulcer, anticancer, and anti‐inflammatory activities (Chen et al. 2020). It is noteworthy that pomegranate flowers exhibit a higher concentration of bioactive compounds in comparison to other plant parts. These include polyphenols, flavonoids, triterpenes, saponins, ellagic acid, gallic acid, ursolic acid, anthocyanins, tricin, oleanolic acid, and daucosterol (Li et al. 2020). Nevertheless, it is essential to evaluate the toxicological impact of these bioactive compounds, as some plant‐derived chemicals may demonstrate toxic characteristics in other organisms (Patel et al. 2008). With regard to the toxicological safety of pomegranate extracts, research findings suggest that pomegranate extracts exhibit relatively low toxicity but high potency. Additionally, it has been reported that toxicity, which is directly correlated with the alkaloid content, is more intense in pomegranate peel and root extracts (Vidal et al. 2003). It is therefore important to consider the potential toxicity of pomegranate extracts when used in food products.

In contrast to the extensive studies on pomegranate fruit, peel, and juice, the present study focuses on the underexplored pomegranate flower as a potential source of natural antioxidants and nitrite substitutes in meat products. Moreover, the present study offers a comparative analysis of the effects of water and ethanol extracts, which has not been sufficiently addressed in previous studies. This study aims to address this lack of knowledge by investigating the potential of PFE as a natural alternative to synthetic additives in meat products.

The objective of the present study was to investigate the potential use of PFE obtained from water and ethanol at antimicrobial doses in heat‐treated chicken sausages. In order to gain a more comprehensive understanding of the potential applications of PFE as a nitrite substitute and their impact on color and sensory properties, the study focused on investigating their effect on chicken sausages, a widely consumed food product. The utilization of both water and ethanol extraction procedures was designed to optimize the recovery of bioactive compounds, given that different solvents are effective in extracting compounds from to different chemical classes. Water, as a natural and nontoxic solvent, was the preferred medium for the extraction of various bioactive compounds, especially those with hydrophilic properties, whereas ethanol was chosen as a solvent to obtain higher concentrations of polyphenol extracts, including those less soluble in water. The present study aimed to investigate the potential impact of PFE on the physicochemical (phenolic content, pH, color, and texture), assays of antioxidant and lipid oxidations, and sensory properties of chicken sausages. Consequently, the impact of PFE on the stability, quality, and consumer acceptance of sausages was determined, and their potential for usage was evaluated.

## 2 Material and Methods

### 2.1 Preparation of Pomegranate Flower Extract (PFE)

In this study, traditionally, Sun‐dried pomegranate flowers grown in the southern region of Turkey (Adana and Mersin provinces) were used. The PFEs were prepared using water and ethanol following the method outlined in our previous study (Özer, Var, and Demir Özer 2021). The extraction parameters were optimized in that study to achieve highest antioxidant capacity and phenolic content, and these optimal conditions were applied in the present extraction process. Briefly, 1 g of pomegranate flower was extracted with 20 mL of solvent (distilled water or ethanol [70%, w/w]) at 40°C for 40 min in a shaking water bath. Following the extraction process, the mixture was passed through a coarse filter paper, after which the solvent was removed by rotary evaporation. The mixture was then lyophilized (Operon, OPR‐FDU‐8612, South Korea) at −80°C and 0.01‐mbar pressure, resulting powdered extracts.

### 2.2 Determination of Minimum Inhibitory Concentration of Extracts

The minimum inhibitory concentration (MIC) for water and ethanol extracts of pomegranate flowers was determined by the agar dilution method as described by Al‐Zoreky (2009). *Escherichia coli* O157:H7 ATTC 43897 was used as the control strain, and sodium nitrite was used as the reference antimicrobial. The dose of sodium nitrite was used up to 150 ppm, which is the highest dose allowed for use in sausage production. In addition, the extract doses (mg/mL) were increased until the dose (mg/mL) was reached that can show the effect of sodium nitrite on *E. coli* O157:H7 ATCC 43897 at a level of 10^5^ log cfu/g. *E. coli* O157:H7 ATCC 43897 was inoculated onto sterilized medium with the extract and sodium nitrite and incubated at 37°C for 48 h. The lowest extract dose (mg/ml) at which no bacterial growth occurred was determined as the MIC dose.

### 2.3 Production of Heat‐Treated Sausage

Chicken breast meat (*Pectoralis major*), beef tallow, and spices ingredients were provided by local suppliers (Nevsehir, Turkey). Sausage production was performed according to the procedure of Kılıç and Özer (2017). The sausage mixture was prepared as 70% chicken breast meat, 22% beef fat, 1.5% sodium chloride, 6.2% spices (garlic, red pepper, cumin, black pepper, allspice, sucrose, and wheat fiber), 0.05% sodium ascorbate, and 0.25% sodium polyphosphate. The amount of sodium nitrite and PFE contained in the treatments was presented in the experimental design (Table 1). An experimental design was established for the purpose of determining the effects of incorporating PFE into products containing only sodium nitrite at a level sufficient for color and microbial stability (50 and 100 ppm, respectively), as well as into products that do not contain sodium nitrite. The control groups, designated as positive control and negative control, were prepared with the addition of 150‐ppm sodium nitrite and without sodium nitrite, respectively. In all other groups, water and ethanol extracts were used at a concentration of 350 and 200 ppm, respectively. The concentrations selected were based on the MICs determined for the extracts, as previously described in Section 2.2. The MIC values indicate the lowest concentrations at which the extracts demonstrated antimicrobial activity against *E. coli* O157:H7, thereby ensuring their efficacy at these levels within the sausage formulation. Moreover, in combination with PFE, sodium nitrite was used at reduced doses in Groups 3 and 4 for 100 ppm and Groups 5 and 6 for 50 ppm. After all the ingredients were mixed, sausage mixture was stuffed into collagen casings. The sausages were then heat treated for 15 min at a minimum internal temperature of 65°C. After cooling to room temperature, the sausages were vacuum‐stored at 4°C for 30 days.

**TABLE 1:** Experimental design for sausage production.

Groups	Sodium nitrite	WE	EE
Positive control	150 ppm	—	—
Negative control	—	—	—
Group 1	—	350 ppm	—
Group 2	—	—	200 ppm
Group 3	100 ppm	350 ppm	—
Group 4	100 ppm	—	200 ppm
Group 5	50 ppm	350 ppm	—
Group 6	50 ppm	—	200 ppm

Abbreviations: EE, ethanol extract of pomegranate flower; WE, water extract of pomegranate flower.

### 2.4 Physicochemical Analysis

The pH of the sausages was measured during production and storage. A 10‐g sample was homogenized with 100 mL of distilled water, and the pH was determined using a calibrated pH meter with buffer solutions. L*, a*, and b* values of the sausage samples were assessed using a colorimeter (Minolta, CR 400, Japan), which was calibrated against a standard plate (D65, L* = 97.79, a* = −0.11, and b* = 2.69). The color analysis was conducted utilizing illuminant D65, a 10° standard observer angle, and an 8‐mm aperture in specular component included (SCI) mode. The fat content of the sausages was determined using the Soxhlet method, protein content using the Kjeldahl method, and the ash and moisture contents using gravimetric methods, following the procedures recommended by AOAC (2010).

### 2.5 Residual Nitrite Analysis

The residual nitrite content of sausages was determined during production and storage using the colorimetric method described in Sen and Donaldson (1978). First, a 10‐g sample was homogenized with 70‐mL distilled water and 12‐mL NaOH solution (2%, w/w). The sample was heated to 50°C, and ZnSO_4_ solution (0.42 M) was added. After 10‐min heat treatment in 50°C, the mixture was cooled to room temperature. The mixture was then diluted with 100‐mL distilled water and filtrated (Whatman No:1). First, 20‐mL filtrate was discarded, and 10‐mL filtrate from the remaining filtrate was mixed with 9‐mL NH_4_Cl buffer, 5‐mL acetic acid (60%) solution, and 10‐mL color reagent (sulfanilic acid and N‐[l‐naphthyl] ethylene diamine). The mixture was diluted into appropriate dilutions and incubated in dark for 25 min. Thereafter, the absorbance of the mixture was determined at 550 nm. The results were calculated from standard curve of nitrite, and results were presented in mg/kg of the sample.

### 2.6 TBARS Analysis

Thiobarbituric acid reactive substance (TBARS) analysis was conducted to assess the oxidative stability of sausages during production and storage period. TBARS values of the samples were measured by the procedure of Kilic and Richards (2003). Briefly, 1 g of the sample was homogenized with 6 mL of trichloroacetic acid (TCA) solution. The mixture was then filtered using Whatman No. 1 filter paper, and 1 mL of the filtrate was collected. To this, 1 mL of thiobarbituric acid (TBA) solution was added, and the mixture was incubated at 100°C for 40 min. After cooling, the mixture was centrifuged at 2000 rpm for 5 min. The absorbance of the samples was measured at 532 nm against a blank using a UV–Vis spectrophotometer. Results were presented in μmol malondialdehyde/kg of the sample.

### 2.7 Assays of Antioxidant Status

The antioxidant activity of the sausage samples was determined by analyzing the methanolic extracts of the samples. The extracts were prepared as defined by Mancini et al. (2015) with minor changes. A sample of 2.5 g was homogenized with 5 mL of ethanol at 5000×*g* for 2 min. The mixture was then separated by centrifugation at 8000×*g* for 20 min, and the filtrate was obtained by filtration (Whatman No. 1). This filtrate obtained was used to measure the DPPH radical scavenging capacity and β‐carotene bleaching activity of sausages.

#### 2.7.1 DPPH Radical‐Scavenging Activity

The DPPH radical scavenging capacity for samples was analyzed using the method outlined by Jung et al. (2010). DPPH solution of 200 μL of extract, 800 μL of distilled water, and 1 mL of 0.2 mM was mixed. The mixture was allowed to stand at room temperature for 30 min. Subsequently, the absorbance for samples was determined at 517 nm in a UV–Vis spectrophotometer. The control was prepared using water and DPPH solution. The antioxidant capacity of the samples was estimated according to the following equation:

$$\text{DPPH activity}=\left[1-\left({\mathrm{Abs}}_{\text{sample}}/{\mathrm{Abs}}_{\text{control}}\right)\right]\times 100.$$

#### 2.7.2 β‐Carotene Bleaching Activity

The β‐carotene bleaching activity of the samples was measured by the procedure of Özer, Var, and Demir Özer (2021). One milliliter of extract was mixed with 3 mL of test solutions (0.002‐g β‐carotene, 40‐μL linoleic acid, and 400‐μL Tween 40 in 20‐mL chloroform), and the absorbance was measured at 417 nm in a UV–Vis spectrophotometer. The mixtures were then incubated at 50°C for 100 min, and the absorbance was detected. The calculation of the β‐carotene bleaching activity was made by the following equation with the difference between the two absorbance values.

$${\mathrm{DR}}_{\text{sample},\text{control}}=\ln\;\left({\mathrm{Abs}}_{\text{first}}/{\mathrm{Abs}}_{\text{incubation}}\right)\times 1/100,$$

$$\beta -\text{carotene bleaching activity}=\left({\mathrm{DR}}_{\text{control}}-{\mathrm{DR}}_{\text{sample}}\right)/{\mathrm{DR}}_{\text{control}}\times 100.$$

### 2.8 The Total Amount of Phenolic Substance

The total amount of phenolic substances was determined by making some modifications to the procedure described by Özer, Var, and Demir Özer (2021). First, 0.5 mL of extract was mixed with 23 mL of distilled water and 0.5 mL of Folin–Ciocalteu reagent. The mixture was kept at room temperature for 3 min. Then, 1.5‐mL sodium carbonate solution (2%, w/v) was added and mixed for 120 min. The absorbance was then detected at 760 nm in a spectrophotometer. The total amount of phenolic substances in the samples was determined as gallic acid equivalent (mg GAE/g sample) by plotting the calibration curve using gallic acid standard.

### 2.9 Texture Analysis

The texture profile analysis was conducted on samples using a Texture Analyzer (TA‐XT Plus, Stable Micro Systems, United Kingdom) equipped with an aluminum pressure probe (*p*/100, 100 mm Ø). Sausages of 50‐mm diameter were sliced to 10‐mm thickness to provide samples for texture analysis (50 mm Ø × 10 mm). The test, pretest, and posttest speed was 1, 3, and 3 mm/s, respectively. A 70% compressive deformation was applied using a 50‐kg load cell. The hardness, cohesiveness, gumminess, springiness, resilience, and chewiness values of the samples were calculated as described by Bourne (1978).

### 2.10 Sensory Analysis

Twelve panelists trained in sensory analysis of foods conducted differences and descriptive sensory analysis using methods defined by the IFT (1981). Panelists attended a training session focused on the identification and evaluation of sensory attributes prior to sensory analysis to facilitate consistency and reliability in their assessments. The sensory analysis was conducted using sausages obtained on the day of production. The sausages were sliced to 10‐mm thickness and were heated at hot‐plate until the internal temperature reached 72°C. Subsequently, samples coded with three randomly generated numbers were served to the panelists while still at the optimal serving temperature (~65°C). In each sensory session, the panelists evaluated the eight different sausage samples. To refresh the palates of panelists, unsalted bread and water were served with sausages. The panelists evaluated to indicate the intensity of external color, cross‐sectional color, texture, odor, taste, aroma, and overall acceptability of the sausages, using a 9‐point scale.

### 2.11 Statistical Analysis

The study was conducted as a fully randomized trial with eight experimental groups and two replicates on separate production days. The data obtained from all analysis were evaluated by SPSS 22.0.0 (IBM SPSS Statistics, United States) with a 95% confidence interval using one‐way ANOVA. When analyzing the data, treatment and storage periods were considered as fixed factors and replications as a random factor in the model. The Duncan post hoc multiple comparison test was used to determine the significance of the difference between the means of the experimental groups (*p* < 0.05). The results were reported as means with their standard deviations. Additionally, sensory, texture profile, and color analysis data were analyzed using principal component analysis (PCA) in Minitab 17 (Minitab Ltd., Coventry, United Kingdom).

## 3 Results and Discussion

Based on the literature, the toxicological potential of water and ethanol extracts used at doses of 350 and 200 ppm, respectively, in sausage production has been evaluated. Studies on rats, mice, and other rodent models have reported that a dose of 600 mg/kg body weight/day represents the no‐observed‐adverse‐effect level (NOAEL) for pomegranate furit extract (Patel et al. 2008). Furthermore, the use of extracts derived from whole pomegranate at a dose of 1.2 mg/kg has been shown to cause no toxic effects (Vidal et al. 2003). Therefore, the extracts used in the sausage formulation are considered to have a low potential for adverse toxicological effects.

**FIGURE 1:**
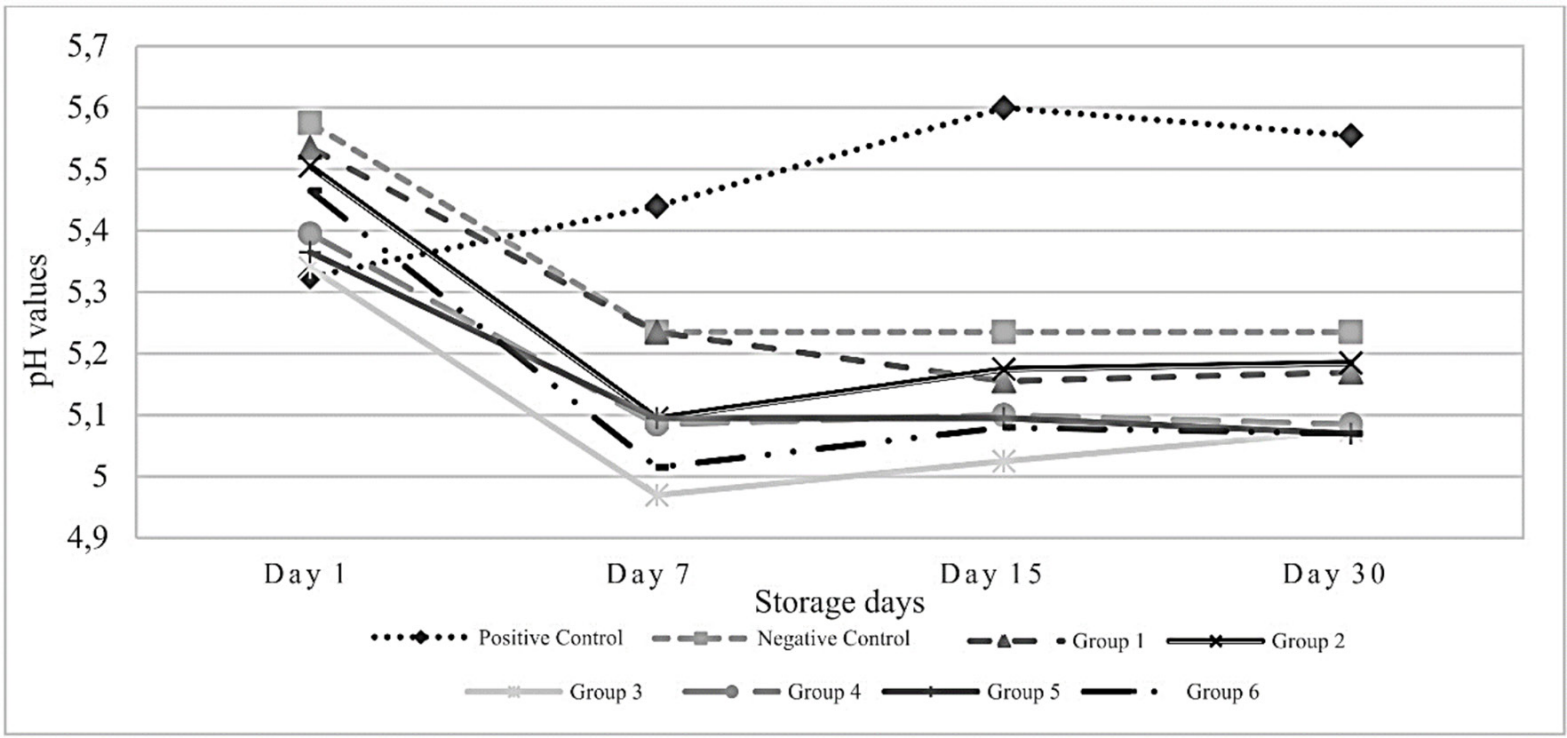
The changes in pH values of sausage samples during storage period. Groups are defined as follows: positive control (150 ppm nitrite), negative control (no nitrite), Group 1 (350 ppm aqueous pomegranate flower extract [PFE]), Group 2 (200 ppm ethanolic PFE), Group 3 (100 ppm nitrite + 350 ppm aqueous PFE), Group 4 (100 ppm nitrite + 200 ppm ethanolic PFE), Group 5 (50 ppm nitrite + 350 ppm aqueous PFE), and Group 6 (50 ppm nitrite + 200 ppm ethanolic PFE).

The use of PFE at different concentrations in the sausage formulation did not result in significant differences in the moisture, ash, protein, and fat contents of sausages as expected (data not presented). In addition, the proximate composition of the control groups, with and without nitrite, was found to be no different from that of the experimental groups containing PFE. The average moisture, ash, protein, and fat contents of the samples were 53.24%, 2.23%, 21.02%, and 23.95%, respectively. Many studies have reported that herbal extracts used in meat formulation do not affect the proximate composition but significant changes in pH values can be observed depending on the dose used (İnce, Efil, and Özfiliz 2022; Khalehgi et al. 2016; Serdaroğlu et al. 2023). The results of pH analysis showed that pomegranate extracts and storage time had significant effects on pH values (*p* < 0.05) (Figure 1). However, no significant differences were found among groups that contain water and ethanolic PFE. There was a significant decrease in pH values with increasing storage time (*p* < 0.05), except for the positive control group containing 150 ppm nitrite. Although there was a significant increase in the pH value of the positive control group up to the 15th day of storage, a significant decrease in the pH value occurred on the 30th day of storage (*p* < 0.05). On the other hand, it was determined that the pH values decreased in all other groups containing PFE. The present results are similar to some studies in the literature. In the study of Firuzi et al. (2019), the pH of samples with pomegranate rind powder extract decreased by 3.21% in frankfurters after 60 days of storage. Similarly, it was reported that the pH for beef patties was considerably affected by both storage and the addition of pomegranate peel extract (Bouarab‐Chibane et al. 2017).

The changes in residual nitrite content of sausage samples during storage period were presented in Table 2. As expected, the highest residual nitrite levels were observed in the positive control sample, which was prepared with the addition of 150‐ppm sodium nitrite. In contrast, the lowest residual nitrite levels were observed in the nitrite‐free groups (negative control, Group 1, and Group 2) (*p* < 0.05). As the addition of sodium nitrite decreased in all other groups, the amount of residual nitrite also decreased (*p* < 0.05). In addition, storage time was an important factor in the reduction of residual nitrite (*p* < 0.05). It is thought that the amount of residual nitrite in cured meat products decreases due to enzymatic and chemical reactions such as oxidation during storage (Jantapirak et al. 2023; Shakil et al. 2022). A comparative analysis of the residual nitrite levels in Group 2 and the PFE‐containing groups revealed that approximately 9.5 mg/kg sample of nitrite is derived from the naturally occurring composition of the meat whereas the incorporation of PFE resulted in a slight increase of approximately 1 mg/kg in the residual nitrite levels. This suggests that nitrite is naturally present in meat and, to a lesser extent, also in the PFE itself. Similar findings have been reported in the literature. For example, Bahadoran et al. (2016) indicated the presence of up to 1.5 and 37 mg/kg of nitrite in pomegranate and meat, respectively. Another important finding was that PFE used with sodium nitrite had no significant effect on residual nitrite levels. However, Viuda‐Martos et al. (2009) reported that polyphenols can reduce the amount of residual nitrite due to their reaction with nitrite. It has been reported that bioactive compounds such as caffeic acid, ferulic acid, hesperidin, and narirutin, which are also found in pomegranate and pomegranate compounds, react readily with nitrite (Poyrazoğlu, Gökmen, and Artιk 2002; Saffarzadeh‐Matin and Khosrowshahi 2017). However, Xie et al. (2019) indicated that the concentration of these components and pH is an important factor for this reaction. Therefore, depending on the PFE concentration used and the pH of sausages, it is thought that the use of PFE does not significantly reduce the residual nitrite content in sausage samples.

**TABLE 2:** The changes in residual nitrite values of sausages during storage.

Groups (mg/kg sample)	Day 1	Day 7	Day 15	Day 30
Positive control	24.27 ± 0.32^aA^	23.79 ± 0.26^aA^	22.48 ± 0.05^aB^	21.29 ± 0.31^aC^
Negative control	9.54 ± 0.04^eA^	9.42 ± 0.07^eA^	9.01 ± 0.21^eB^	8.66 ± 0.33^fB^
Group 1	10.62 ± 0.03^dA^	10.51 ± 0.13^dA^	10.25 ± 0.05^dB^	10.01 ± 0.07^dB^
Group 2	10.43 ± 0.06^dA^	10.28 ± 0.06^dA^	9.91 ± 0.08^eB^	9.71 ± 0.09^eB^
Group 3	16.40 ± 0.16^bA^	16.18 ± 0.14^bA^	14.68 ± 0.04^bB^	13.53 ± 0.04^bB^
Group 4	16.67 ± 0.18^bA^	16.24 ± 0.16^bA^	14.92 ± 0.11^bB^	13.78 ± 0.06^bB^
Group 5	12.88 ± 0.04^cA^	12.74 ± 0.03^cA^	11.81 ± 0.12^cB^	11.09 ± 0.03^cB^
Group 6	12.81 ± 0.02^cA^	12.69 ± 0.13^cA^	12.04 ± 0.12^cB^	11.51 ± 0.17^cB^

*Note:* a, b, c ↓ means followed by different small letters in the same column are significant (*p* < 0.05). A, B, C → means followed by different capital letters in the same row are significant (*p* < 0.05). Groups are defined as follows: positive control (150 ppm nitrite), negative control (no nitrite), Group 1 (350 ppm aqueous pomegranate flower extract [PFE]), Group 2 (200 ppm ethanolic PFE), Group 3 (100 ppm nitrite + 350 ppm aqueous PFE), Group 4 (100 ppm nitrite + 200 ppm ethanolic PFE), Group 5 (50 ppm nitrite + 350 ppm aqueous PFE), Group 6 (50 ppm nitrite + 200 ppm ethanolic PFE).

The changes in TBARS values of sausage samples during storage periods were given in Table 3. The TBARS analysis results indicate that no differences among treatment groups were found between the 1st and 7th day, whereas significant differences were found between the 15th and 30th day (*p* < 0.05). Also, the TBARS values for all groups increased during the storage period (*p* < 0.05). The results indicated that the use of PFE had a very significant effect on TBARS values and resulted in a decrease in TBARS values after the 15th day of storage (*p* < 0.05). On the 15th day of storage, the TBARS levels of the groups that used PFE along with 100‐ and 50‐ppm sodium nitrite were the same as those of positive control (150‐ppm sodium nitrite). However, on the last day of storage, the TBARS values for the groups that used PFE together with 50‐ppm sodium nitrite were considerably lower than the other treatments (*p* < 0.05). The findings demonstrate a strong correlation between the PFE and the lipid oxidation inhibitory activity of sodium nitrite at low doses. Numerous studies have demonstrated that incorporating pomegranate products into meat products enhances their oxidation stability. Lyophilized pomegranate peel nanoparticles (LPP‐NPs) in meatballs (Morsy, Mekawi, and Elsabagh 2018), pomegranate peel powder in beef sausage (El‐Nashi et al. 2015), pomegranate peel powder extract, pomegranate juice and pomegranate seed powder extract in raw pork meat (Qin et al. 2013), and pomegranate peel extract in frankfurter (Firuzi et al. 2019) were proved to inhibit the oxidation reactions. However, in these studies, the interaction of pomegranate components with nitrite and its consequences on oxidation were not examined. Present results show that PFE used at antimicrobial doses are not as strong as sodium nitrite for antioxidant effect alone, but their use together with low doses of nitrite may have significant antioxidant potential. Such an effect is rarely observed with other plant extracts in meat products, thereby underscoring the potential of PFE to reduce nitrite levels while preserving the safety and quality of the final product. On the other hand, the ineffectiveness of using PFE without nitrite may be due to the degeneration of antioxidant components caused by heat applied during sausage production. On the other hand, the effectiveness of using PFE with nitrite may be related to the reaction of components such as punicalagin and ellagic acid, which are abundant in pomegranate and its by‐products, with nitrite. Some studies reported that when phenolic components and nitrite are used together, they may have an antagonist or even prooxidant effect on antioxidant activity (Hayes, Canonico, and Allen 2013; Khalehgi et al. 2016). Xie et al. (2019) also indicated that polyphenolic compounds in pomegranate peel react with nitrite, forming nitroso compounds, particularly at low pH values (pH < 4.5). For these reasons, the effectiveness of the phenolic compounds and nitrite is significantly affected by their concentration due to its providing pH decreases.

**TABLE 3:** The changes in TBARS values of sausages during storage.

Groups	Day 1	Day 7	Day 15	Day 30
Positive control	2.01 ± 0.01^bC^	2.00 ± 0.09^bC^	3.31 ± 0.08^bB^	6.26 ± 0.15^cA^
Negative control	2.05 ± 0.07^abC^	2.10 ± 0.14^abC^	4.46 ± 0.03^aB^	8.43 ± 0.05^aA^
Group 1	2.02 ± 0.03^bC^	2.05 ± 0.07^abC^	4.11 ± 0.23^aB^	7.62 ± 0.21^bA^
Group 2	2.03 ± 0.01^bC^	2.46 ± 0.15^aC^	4.25 ± 0.36^aB^	7.93 ± 0.11^abA^
Group 3	2.10 ± 0.12^abC^	2.17 ± 0.36^abC^	3.04 ± 0.00^bB^	5.40 ± 0.36^dA^
Group 4	2.07 ± 0.08^abC^	2.22 ± 0.22^abC^	3.14 ± 0.27^bB^	5.79 ± 0.31^cdA^
Group 5	2.07 ± 0.07^abC^	2.06 ± 0.01^abC^	2.84 ± 0.00^bB^	5.37 ± 0.01^dA^
Group 6	2.20 ± 0.01^aC^	2.15 ± 0.05^abC^	3.02 ± 0.24^bB^	5.61 ± 0.32^dA^

*Note:* a, b, c ↓ means followed by different small letters in the same column are significant (*p* < 0.05). A, B, C → means followed by different capital letters in the same row are significant (*p* < 0.05). Experimental groups are defined as follows: positive control (150 ppm nitrite), negative control (no nitrite), Group 1 (350 ppm aqueous pomegranate flower extract [PFE]), Group 2 (200 ppm ethanolic PFE), Group 3 (100 ppm nitrite + 350 ppm aqueous PFE), Group 4 (100 ppm nitrite + 200 ppm ethanolic PFE), Group 5 (50 ppm nitrite + 350 ppm aqueous PFE), Group 6 (50 ppm nitrite + 200 ppm ethanolic PFE).

**FIGURE 2:**
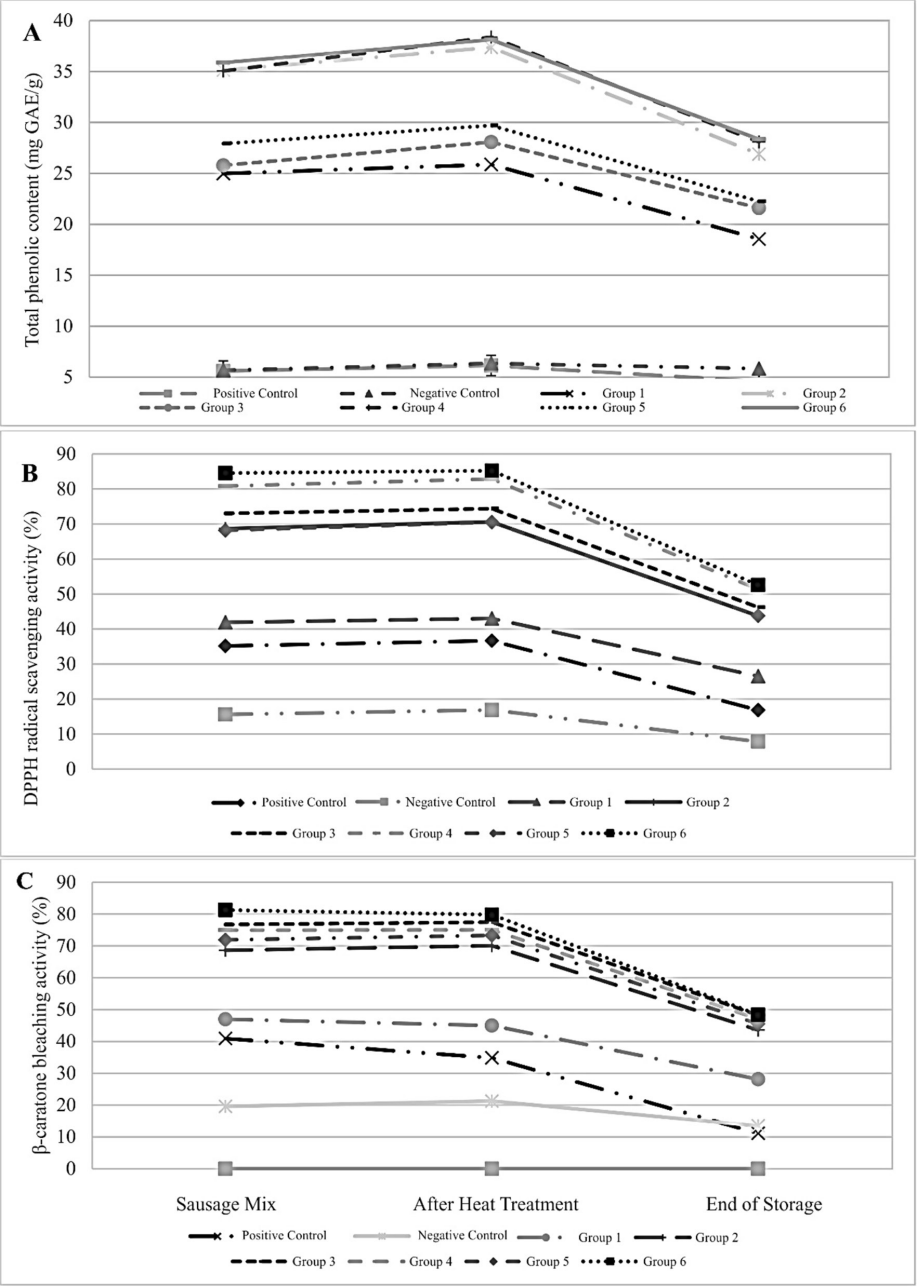
The changes in total phenolic content and antioxidant capacity of sausages. (A) Total phenolic content (mg GAE/g); (B) DPPH radical scavenging activity (%), 30 min, 100 μg/mL; (C) β‐carotene bleaching activity (%), 100 min, 100 μg/mL. Groups are defined as follows: positive control (150 ppm nitrite), negative control (no nitrite), Group 1 (350 ppm aqueous pomegranate flower extract [PFE]), Group 2 (200 ppm ethanolic PFE), Group 3 (100 ppm nitrite + 350 ppm aqueous PFE), Group 4 (100 ppm nitrite + 200 ppm ethanolic PFE), Group 5 (50 ppm nitrite + 350 ppm aqueous PFE), and Group 6 (50 ppm nitrite + 200 ppm ethanolic PFE).

The changes in antioxidant capacity (DPPH radical scavenging and β‐carotene bleaching activity) and total phenolic contents of sausage samples were presented in Figure 2. The results showed that PFE type and sodium nitrite concentration significantly affected the antioxidant capacity of sausage samples (*p* < 0.05). The sausages containing PFE exhibited higher DPPH radical scavenging activity compared to those containing only sodium nitrite (positive control) (*p* < 0.05). The group that only contained the ethanolic extract of pomegranate flower exhibited twice the DPPH radical scavenging activity compared to the group that only contained 150 ppm sodium nitrite (*p* < 0.05). Additionally, the DPPH radical scavenging activity of samples prepared with ethanol extract of pomegranate flower was found to be significantly higher than that of water extracts (*p* < 0.05). The highest DPPH radical scavenging activity was detected in Group 6 containing 200‐ppm PFE and 50‐ppm nitrite (*p* < 0.05). Furthermore, it was observed that the DPPH activity value increased when nitrite and extracts were used together whereas the DPPH activity values of all groups decreased during storage. On the other hand, the sausages containing nitrite and PFE exhibited a similar trend in β‐carotene bleaching activity as in DPPH radical scavenging activity. The sausages containing ethanolic PFE showed significantly higher β‐carotene bleaching values compared to those containing aqueous PFE (*p* < 0.05). Additionally, β‐carotene bleaching values increased when nitrite and extracts were used together (*p* < 0.05).

As predicted, the addition of PFE significantly increased the total phenolic compound content of the sausages (*p* < 0.05). The total phenolic content of the sausage is consistent with the antioxidant capacity obtained. The study found that sausages containing both 200‐ppm ethanolic PFE and 50‐ppm sodium nitrite had a higher total phenolic content compared to the other groups throughout all production stages and at the end of the storage period (*p* < 0.05). However, the total phenolic content of all sausage samples decreased during storage (*p* < 0.05). The antioxidant activity values and total phenolic content obtained in the study are similar to many studies in the literature (Das et al. 2021; Esfahani et al. 2022; Fourati et al. 2020; Qin et al. 2013). Pomegranate peel extracts were found to have high antioxidant, and free radical scavenging activities can inhibit oxidation in ground buffalo meat by Ghimire, Paudel, and Poudel (2022). In a study on chicken meatballs, the use of peel and extracts from pomegranate was found to increase the total phenolic content of the meatballs (Sharma and Yadav 2020). Panza, Conte, and del Nobile (2021) indicated that pomegranate peel powder exhibited high levels of phenolic and antioxidant activity in ready‐to‐cook cod sticks coated with pomegranate peel powder. Another study reported that pomegranate peel extract caused an increase in total phenolics and antioxidant capacity in cooked chicken meatballs (Naveena, Sen, Kingsly, Singh, and Kondaiah 2008).

**FIGURE 3:**
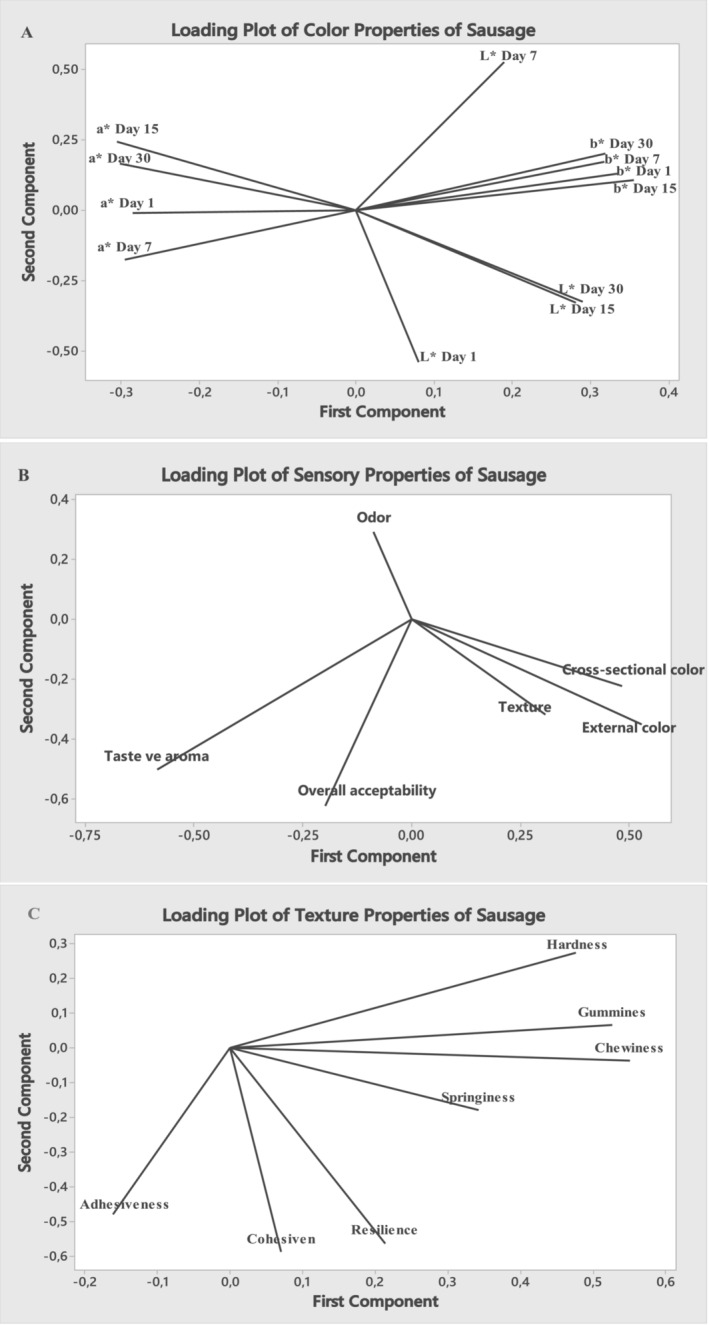
Principal component analysis (PCA) of all groups, plot of the load distribution of the color (A), sensory (B), and texture (C) properties during storage. The *X* axis represents the first principal component, and the *Y* axis represents the second principal component. The correlation between variables is indicated by their position on the graph; variables in close proximity have similar properties, whereas those at a greater distance apart exhibit differences.

The color scores (Lab* values) of heat‐treated chicken sausages were examined by the principal component analysis (PCA) (Figure 3A). The results showed that there was a positive correlation between the 30th day L* and the 15th day L* (*r* = 0.992, *p* ≤ 0.01). During storage, a* and b* values showed a positive correlation and the correlation coefficients varied between 0.592–0.973 and 0.855–0.939, respectively (*p* ≤ 0.01). However, during storage, a negative correlation was found between the b* and a* values (*p* ≤ 0.01). The Pearson correlation coefficient values between all a* values and all L* values during storage are negative. On the other hand, the Pearson correlation coefficient between all b* values and L* values is positive (Ijaz et al. 2020).

According to the results of the color analysis, there were no significant differences in the L* value among the groups containing PFE and the control groups. The use of sodium nitrite at a dose of 150 ppm significantly increased a* values and decreased b* values of sausages (*p* ≤ 0.05). Conversely, as a result of the use of PFE, even PFE use with a reduced dose of sodium nitrite (100 and 50 ppm), the a* and b* values of the samples were not as high and low as in positive control group, respectively. The results indicate that aqueous and ethanol extracts of pomegranate flower and sodium nitrite at reduced dose are not as effective as sodium nitrite on the color parameter of heat‐treated chicken sausages. Similarly, the use of sodium nitrite at doses of 75 and 50 ppm had a negative effect on the color values of minced roasted beef samples (Wójciak, Stasiak, and Kęska 2019). Naveena, Sen, Vaithiyanathan, Babji, and Kondaiah (2008) indicated that pomegranate juice did not have the expected effect on the color properties of cooked chicken patties.

PCA was performed to discriminate the sensory properties (exterior color, cross sectional color, odor, taste and aroma, texture, and overall acceptability) of heat‐treated chicken sausages with the addition of PFE (Figure 3B). A statistically significant positive correlation was found between overall acceptability and taste and aroma when the loading plot score was examined (*r* = 0.240, *p* < 0.01). In addition, it was also found that there was a positive correlation that was also found between overall acceptability and external color, internal section color, and texture. Cross‐sectional color was found to correlate positively with external color (*r* = 0.393, *p* < 0.01). However, a negative correlation was observed between odor and both external and cross‐sectional color (*p* > 0.05). Texture showed a positive correlation with all groups, but only the correlation between texture and external and cross‐sectional color was deemed statistically significant (*r* = 0.272, 0.242, *p* < 0.01, respectively). Furthermore, there is a positive correlation between taste and aroma, external color and cross‐section color, and a negative correlation between odors. In addition to all these, although the positive control group was found to be more preferable in terms of exterior color, texture, and overall acceptability properties, it was stated that the sausages containing PFE were generally at an acceptable level.

The textural attributes of hardness, cohesiveness, springiness, chewiness, gumminess, and resilience were investigated in heat‐treated chicken sausages (Figure 3C). The relationship between the textural properties of heat‐treated chicken sausages was evaluated with Pearson correlation coefficients. PCA results showed that hardness and adhesiveness were strongly correlated (*p* < 0.05) (Herrero et al. 2008). As the adhesiveness score increased, the hardness score decreased, and a negative correlation was observed (*r* = −0.456, *p* < 0.05). As the chewability score increased, the hardness (*r* = 0.792, *p* < 0.01), springiness (*r* = 0.722, *p* < 0.01) and gumminess (*r* = 0.888, *p* ≤ 0.01) scores increased, and a positive correlation was found between the groups. It was also found that there was a positive correlation between the gumminess and hardness (*r* = 0.934, *p* ≤ 0.01) scores. It was also observed that as the resilience score increased, the cohesiveness (*r* = 0.878, *p* ≤ 0.01) and adherence (*r* = 0.500, *p* ≤ 0.01) scores also increased. Similarly, studies in the literature have reported that chewiness and gumminess scores are directly proportional to hardness and that there is an increase in chewiness and gumminess scores in meat products (Ghanghas 2023; Şayin Sert and Coşkun 2022).

The texture results indicated that the positive control group, which contained only nitrite, had the lowest values for cohesiveness and elasticity. However, no significant differences were found between negative control and the pomegranate extract‐added groups, which had higher values than positive control. Numerous studies have reported that the use of sodium nitrite in sausages reduces cohesiveness and firmness by directly or indirectly affecting many parameters, especially pH and moisture (Dong et al. 2007; Gimeno et al. 2000). Therefore, to avoid negative correlations between sodium nitrite dose and textural parameters, the combined use of low doses of nitrite and PFE may be an important alternative.

The present study demonstrates that PFE can effectively enhance nitrite‐reduced chicken sausages' oxidative stability and bioactive content. The findings of the present study provide considerable evidence in support of the potential of PFE as a natural nitrite substitute, particularly in enhancing oxidative stability and bioactive content in processed meat products. Nevertheless, comprehensive toxicological assessments are required to validate its safety for long‐term consumption and its suitability for widespread application. Although the doses used align with reported safety levels, the long‐term effects of regular consumption and potential interactions with other food components require further investigation. It is therefore recommended that further research be carried out to address these gaps in order to ensure the safe and effective use of PFE in meeting consumer demands for healthier and more natural food additives.

## Conflicts of Interest

The authors declare no conflicts of interest.

## Supporting information


**Figure S1** The changes in a* and b* values for sausage samples during storage.

**Table S1** The texture profile analysis results of sausages.

**Figure S2** Sensory analysis results of heat‐treated sausages with pomegranate flower extract.
