# Supplementary material for: Effects of Pomegranate Flower Extracts on Antioxidant Properties, Phenolic Content, and Quality Attributes of Nitrite Reduced Chicken Sausages
Source: Anim Sci J. 2025 Feb 13;96(1):e70039. doi: 10.1111/asj.70039 (PMC11825183; doi:10.1111/asj.70039)
Supplement: Supplementary file 1 — Figure S1 The changes in a* and b* values for sausage samples during storage. [file ASJ-96-e70039-s003.docx]

|  |  | Positive control | Negative control | Group 1 | Group 2 | Group 3 | Group 4 | Group 5 | Group 6 |  |
| --- | --- | --- | --- | --- | --- | --- | --- | --- | --- | --- |
| Storage days | 1 |  |  |  |  |  |  |  |  | a* values |
|  | 7 |  |  |  |  |  |  |  |  |  |
|  | 15 |  |  |  |  |  |  |  |  |  |
|  | 30 |  |  |  |  |  |  |  |  |  |
|  | 1 |  |  |  |  |  |  |  |  | b* values |
|  | 7 |  |  |  |  |  |  |  |  |  |
|  | 15 |  |  |  |  |  |  |  |  |  |
|  | 30 |  |  |  |  |  |  |  |  |  |

Figure. The changes in a* and b* values for sausage samples during storage

*Groups are defined as follows: Positive Control (150 ppm nitrite), Negative Control (no nitrite), Group 3 (100 ppm nitrite + 350 ppm aqueous pomegranate flower extract (PFE)), Group 4 (100 ppm nitrite + 200 ppm ethanolic PFE), Group 5 (50 ppm nitrite + 350 ppm aqueous PFE), Group 6 (50 ppm nitrite + 200 ppm ethanolic PFE).
