# Supplementary material for: Effects of Pomegranate Flower Extracts on Antioxidant Properties, Phenolic Content, and Quality Attributes of Nitrite Reduced Chicken Sausages
Source: Anim Sci J. 2025 Feb 13;96(1):e70039. doi: 10.1111/asj.70039 (PMC11825183; doi:10.1111/asj.70039)
Supplement: Supplementary file 2 — Table S1 The texture profile analysis results of sausages. [file ASJ-96-e70039-s001.docx]

| Groups | Hardness | Springiness | Cohesiveness | Gumminess | Chewiness | Resilience |
| --- | --- | --- | --- | --- | --- | --- |
| Positive Control | 13,63±0,97^cd^ | 0,91±0,04^ab^ | 0,59±0,01^d^ | 8,06±0,45^d^ | 6,52±0,62^b^ | 0.18±0,01^d^ |
| Negative Control | 14,84±1,37^bcd^ | 0,86±0,07^ab^ | 0,63±0,02^bc^ | 9,35±0,65^c^ | 7,93±0,91^ab^ | 0,21±0,01^bc^ |
| Group 1 | 17,67±0,91^a^ | 0,87±0,08^ab^ | 0,62±0,01^c^ | 10,95±0,50^a^ | 9,16±1,29^a^ | 0,20±0,01^c^ |
| Group 2 | 15,74±0,54^ab^ | 0,80±0,03^b^ | 0,63±0,01^bc^ | 9,90±0,20^abc^ | 7,94±0,34^ab^ | 0,21±0,01^bc^ |
| Group 3 | 14,28±1,21^bcd^ | 0,80±0,80^b^ | 0,67±0,01^a^ | 9,50±0,64^c^ | 7,65±1,24^ab^ | 0,22±0,01^ab^ |
| Group 4 | 16,37±1,21^ab^ | 0,83±0,03^ab^ | 0,65±0,01^ab^ | 10,69±0,57^a^ | 9,30±0,33^a^ | 0,23±0,01^a^ |
| Group 5 | 13,36±1,85^d^ | 0,85±0,03^ab^ | 0,67±0,02^a^ | 8,89±0,96^cd^ | 7,70±0,92^ab^ | 0,22±0,01^a^ |
| Group 6 | 15,48±0,44^bcd^ | 0,91±0,03^a^ | 0,65±0,01^ab^ | 10,02±0,29^ab^ | 9,15±0,54^a^ | 0,22±0,01^abc^ |

Table. The texture profile analysis results of sausages

a,b,c ↓ Means followed by different small letters in the same column are significant (P<0.05).

*Groups are defined as follows: Positive Control (150 ppm nitrite), Negative Control (no nitrite), Group 3 (100 ppm nitrite + 350 ppm aqueous pomegranate flower extract (PFE)), Group 4 (100 ppm nitrite + 200 ppm ethanolic PFE), Group 5 (50 ppm nitrite + 350 ppm aqueous PFE), Group 6 (50 ppm nitrite + 200 ppm ethanolic PFE).
