# Supplementary material for: Effects of Pomegranate Flower Extracts on Antioxidant Properties, Phenolic Content, and Quality Attributes of Nitrite Reduced Chicken Sausages
Source: Anim Sci J. 2025 Feb 13;96(1):e70039. doi: 10.1111/asj.70039 (PMC11825183; doi:10.1111/asj.70039)
Supplement: Supplementary file 3 — Figure S2 Sensory analysis results of heat‐treated sausages with pomegranate flower extract. [file ASJ-96-e70039-s002.docx]

Figure . Sensory analysis results of heat-treated sausages with pomegranate flower extract

Groups are defined as follows: Positive Control (150 ppm nitrite), Negative Control (no nitrite), Group 3 (100 ppm nitrite + 350 ppm aqueous pomegranate flower extract (PFE)), Group 4 (100 ppm nitrite + 200 ppm ethanolic PFE), Group 5 (50 ppm nitrite + 350 ppm aqueous PFE), Group 6 (50 ppm nitrite + 200 ppm ethanolic PFE).
